# Danish Nationwide Data Reveal a Link between Diabetes Mellitus, Diabetic Retinopathy, and Glaucoma

**DOI:** 10.1155/2016/2684674

**Published:** 2016-10-31

**Authors:** Anna Horwitz, Beáta Éva Petrovski, Christian Torp-Pedersen, Miriam Kolko

**Affiliations:** ^1^Department of Neuroscience and Pharmacology, University of Copenhagen, Blegdamsvej 3, 2200 Copenhagen, Denmark; ^2^Center for Healthy Aging, University of Copenhagen, Blegdamsvej 3, 2200 Copenhagen, Denmark; ^3^Department of Public Health, University of Szeged, Dóm Tér 10, Szeged 6720, Hungary; ^4^Akershus University Hospital and Institute of Clinical Medicine, University of Oslo, Campus Ahus, 1478 Lørenskog, Norway; ^5^Aalborg University Hospital, Department of Health, Science and Technology, Niels Jernes Vej 12, 9220 Aalborg, Denmark; ^6^Zealand University Hospital, Department of Ophthalmology, Vestermarksvej 23, 4000 Roskilde, Denmark

## Abstract

*Aims*. To determine the association between treatment against diabetes mellitus (DM) and treatment with antiglaucomatous drugs in the entire Danish population and to investigate the comorbidity between DM and its complications with antiglaucomatous treatment.* Methods*. Retrospective nationwide cohort study with data over a 16-year follow-up period. The National Danish Registry of Medicinal Products Statistics was used to identify all claimed prescriptions for antiglaucomatous medication and DM drugs. ICD-10 classifications were furthermore used to identify comorbidities between antiglaucomatous medication and the DM complications, diabetic retinopathy (DR), and nephropathy.* Results*. A total of 6,343,747 individuals in the period between 1996 and 2012 were analyzed. The overall incidence rate of new-onset glaucoma patients was 0.07 per 1000 person-years for the reference population compared to 36 per 1000 person-years for all diagnosed DM cases. Patients treated with DM drugs had about two times higher relative risk of glaucoma, when adjusting for a range of factors. The presence of DR alone or in combination with nephropathy increased the risk of glaucoma.* Conclusions*. The present study reports a strong association between DM and onset of glaucoma treatment in the entire Danish population.

## 1. Introduction

Diabetic retinopathy (DR) is the most common late complication of diabetes mellitus (DM) in the working-age population and one of the leading causes of blindness in the elderly, accounting for a significant drop in quality of life (QoL) and working ability for the patients [1–3]. Nonproliferative DR presents clinically as superficial retinal hemorrhages, cotton wool spots, or microvascular abnormalities [4–6]. Even so, proliferative diabetic retinopathy (PDR) can remain asymptomatic for a very long time [6], and in that light, patients with DM in Denmark are therefore monitored closely by ophthalmologists annually.

The lack of oxygen in the retina causes fragile blood vessels to grow into the vitreous body and along the retina, causing an eminent risk of bleeding and formation of fibrovascular/proliferative membranes, leading consequently to tractional retinal detachment. These new blood vessels can furthermore grow into the angle of the anterior chamber and cause neovascular glaucoma [5].

To date, numerous screening studies have addressed the question of whether DM is a risk factor for primary open angle glaucoma (POAG); however, no converging or uniform conclusions exist to date. Some studies state that POAG is more prevalent in diabetic than in nondiabetic populations [7–12], while others found no statistically increased correlation between the two, although being based on small population sizes and yet raising the main reasonable question concerning patient referral and bias associated with it [9, 13–15]. A recent cohort study from the Tayside region of Scotland included a population of 175,211 participants over a 2-year period and found a relative risk of 1.57 for POAG and 1.38 for elevated eye pressure compared to the rest of the population, although the results were not statistically significant [16].

The aim of the present study was to use data from the entire Danish population using redeemed prescription on antidiabetic and antiglaucoma drugs over a 16-year period and check whether DM is a risk factor for developing glaucoma after adjusting for diabetic complications and several other demographic factors. Furthermore, we wanted to investigate whether any difference exists in glaucoma with regard to medication type, diabetic complications, or concomitant medications such as antihypertensive drugs in patients having DM.

To the best of our knowledge, only one previous cohort study on the topic has been carried out to date, and no study has addressed the association between the medication used within the group of DM patients and patients treated with antiglaucomatous medication. Moreover, the large dataset, amounting to a 16-year follow-up of more than 6 million individuals, constitutes a comprehensive source of data for investigating comorbidities between DM and glaucoma.

## 2. Methods

### 2.1. Registers and Study Population

The study population comprised all individuals living in Denmark in the period between 1996 and 2012 and without previously diagnosed DM or glaucoma, amounting to 6,343,747 individuals. Data from the National Danish Civil Registration System contain information on vital status of all individuals born in, or migrating to, Denmark [17, 18]. In a subpart of the analysis, the sample was restricted to individuals only prescribed antidiabetic medication(s) and, within that group, patients aged 40 to 100 years, thus focusing on age-dependent rather than congenital disorders. The healthcare system in Denmark is fully tax financed and equally available to all inhabitants independent of social and financial status, and the Danish government is responsible for collecting this high-quality, nationwide healthcare data.

The information contained in the database contains dates of redemption of antidiabetic or antiglaucomatous medication (if any) for each individual and furthermore data on patients diagnosed with diabetic complications such as DR and/or nephropathy. The diabetic complications were identified based on the diagnostic codes, according to the International Statistical Classification of Diseases and Related Health Problems (ICD-10), and were retrieved from the register accordingly. On the other hand, the incident glaucoma and DM were based upon the pharmacotherapy used, and glaucoma and DM information was retrieved from the Danish Register of Medicinal Product Statistics, which holds data on all prescriptions dispensed in Denmark that are classified according to the Anatomical Therapeutic Classification system; this register is directly linked to the government for reimbursement purposes and is therefore very accurate. All pharmacies in Denmark are required by the government-financed Danish healthcare system to register all redeemed prescriptions at the individual level by the Danish Personal Identification number (the so-called CPR-number). We hereby identify individuals taking antidiabetic therapy through the National Danish Registry of Medicinal Products Statistics. Drugs administered during a hospital admission are, however, not included. Our data contain the dates of all redeemed antidiabetic treatments as well as all antiglaucomatous treatments, excluding those from before 1995, when mixed data on new and old prescriptions was combined in the register. To assure that the data only contain new prescriptions, all prescriptions registered in the database after January 1, 1996, were therefore included in the study.

### 2.2. Definition of Pharmacotherapy and Comorbidity

*DR* was identified using ICD-10 and diagnosed as DH36, DH368, DH360H, DH360J, DH360K, DH368D, DH368D1, and DH368D2.

*Diabetic nephropathy* was identified using ICD-10 and diagnosed as DE103, DE112, DE132, DE142, DN083, DN083, DN251, and DN.

*Glaucoma*, for the present study, was identified according to the following ACT codes for glaucoma medication: use of *β*-blockers (S01ED01-05); prostaglandin analogues (S01EE); *α*2-adrenergic agonists (S01EA); parasympathomimetic drugs (S01EB); carbon anhydrase inhibitors (S01EC); fixed combination drugs (S01EA51, S01EB51, and S01ED51). Patients were defined as having glaucoma if they received at least 2 prescriptions within 90 days for at least one type of antiglaucomatous medication.

*Hypertension as comorbidity* was used in a subset of the analysis. Hypertension is most often managed by patients' primary physicians, and so the in-hospital ICD-10 diagnosis for essential hypertension (ICD-10I10) is used irregularly. Therefore, identification of hypertensive patients in the studied population was validated by an algorithm (with a positive predictive value of 80.0% and sensitivity of 94.7%), based upon the use of at least 2 classes of antihypertensive drugs (*β*-blockers (BB), renin-angiotensin system (RAS) inhibitors, calcium antagonists (Ccb), or diuretics (DD) and vasoprotectives (VP)) [19]. Patients treated with all drugs types were excluded from the study due to lack of relevant controls.

The ATC codes for antihypertensive drugs used in the analyses are listed here by classes of origin: BB are in the group of C07; RAS inhibitors are in the group of C09; DD are in the group of C03; Ccb are in the group of C08D, and vasoprotectives are in the group of C02. To investigate the effect of pharmacotherapy, the focus was placed on RAS and BB, compared to the rest.

Patients were classified as incident with glaucoma (having their onset of glaucoma) by their first redemption of an antiglaucoma medication and incident with hypertension (having their onset of hypertension) by their first redemption of a second antihypertensive drug.

**Figure 1:**
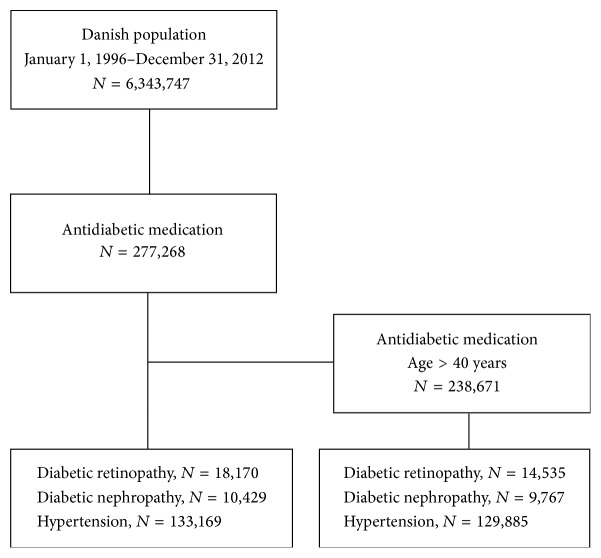
Flowchart of diabetes mellitus and glaucoma in the Danish population in the period from 1996 to 2012. The National Danish Registry of Medicinal Products Statistics was used to identify all individuals who were treated with glaucoma medication and/or antidiabetic drugs.

The selection process for the study population is illustrated in Figure 1.

### 2.3. Rationale for Definitions

DM and glaucoma are most often managed and diagnosed by patients' primary physicians and an out-of-hospital specialist in ophthalmology, respectively. Therefore, the in-hospital ICD-10 diagnoses for the two diseases are not relevant. Instead, we identify the population of glaucoma patients and those with DM and hypertension using the National Danish Registry of Medicinal Products Statistics as descripted above. With this approach, we are able to identify patients with a redeemed prescription for DM and/or glaucoma. Furthermore, this procedure allows us to identify the onset of the condition.

In patients who redeemed antidiabetic medication, comorbidity with hypertension was then identified using a validated algorithm based on the use of at least two classes of antihypertensive drugs. This algorithm is shown to have a tremendous sensitivity of 94.7% and a positive predictive value of 80.0% [19], meaning that our validation algorithm precisely identifies individuals with hypertension.

The Danish Civil Registration System contains information about dates of birth and death of all Danish citizens since 1972 [18], while the Danish Registry of Medicinal Products Statistics contains data on all prescriptions dispensed in Denmark since 1995, including information about size of doses, quantity dispensed, and dispensing date. Prescriptions are classified according to the Anatomical Therapeutic Chemical (ATC) System [17].

### 2.4. Statistical Analysis

To describe the evolution of the incidence of DM, DR, and glaucoma, the incidence rates were calculated in 5-year age strata, as a function of time, and then summarized as events per 1,000 person-years at risk. Furthermore, duration analysis models, based on the Poisson distribution, were employed to investigate the associations between DM treatments and DR, as well as the risk of developing glaucoma, adjusted for a range of potentially confounding factors.

The baseline characteristics are presented as means with standard deviations or frequencies and percentages accordingly. DM was considered to be a time-dependent variable and thus subjects who developed DM contributed risk time in the reference group until the time of diagnosis. Comorbidity was updated continuously throughout the follow-ups. Hazard ratios (HRs) for the study endpoint were estimated using Cox proportional hazards models adjusted for confounding factors including age, sex, comorbidity with DR, hypertension (concomitant medications with antihypertensive drug(s)), and diabetic nephropathy.

The primary analysis was not adjusted for medications used for the treatment of hypertension. An additional analysis was carried out with inclusion of antihypertensives when investigating all patients redeemed with antidiabetic medication to estimate its impact on the HRs of glaucoma. All statistical analyses were performed using SAS 9.4. Heteroscedasticity-robust standard errors were used in the duration model and cluster-robust standard errors, clustered on the individual level, were used in the regression discontinuity models. A significance level of 0.05 was applied, meaning that estimated coefficients with *p* < 0.05 were considered statistically significant and 95% confidence intervals were also reported.

### 2.5. Definitions

The* incidence* in a given year is defined as the number of new cases in that year divided by the number of individuals living in that year. The* incidence rate* is the number of new cases over the 16-year study period per population at risk (measured in 100 person-years). The* relative risk* (RR) of developing glaucoma is the probability of developing glaucoma for a certain group (e.g., males or individuals with DM) divided by the probability of developing glaucoma for the converse group. The estimates of the duration analysis are converted to RR estimates by calculating their antilogarithm.

### 2.6. Outcome

The primary outcome in the present study was glaucoma (as inferred by antiglaucomatous drug prescriptions used).

### 2.7. Ethical Aspects

The Danish Data Protection Agency approved the study (2007-58-0015, int. ref: GEH-2010-001). Retrospective register-based studies do not require ethical approval in Denmark.

## 3. Results

### 3.1. Baseline Characteristics of the Studied Population

The study comprised a total of 6,343,747 subjects within a sixteen-year follow-up. During the study period, 275,078 subjects with incident DM, 75,022 subjects with incident glaucoma, and 18,170 subjects with DR were identified, as shown in the flowchart of the study population selection (Figure 1). The average age at onset for DM was 59.19 years (range: 1.42 to 109.57 years), for DR 56.87 years (range: 4.99 to 98.74 years), and for glaucoma 69.31 years (range: 2.01 to 105.07 years). Median follow-up time was 15.66 (SD 3.08) years and 15.86 (SD 3.33) years for the reference population and DM, respectively. The mean duration from diagnosis of DM to incidence of glaucoma was 4.1 (SD 3.51) years.

### 3.2. Incidence of DM, DR, and Glaucoma

**Figure 2:**
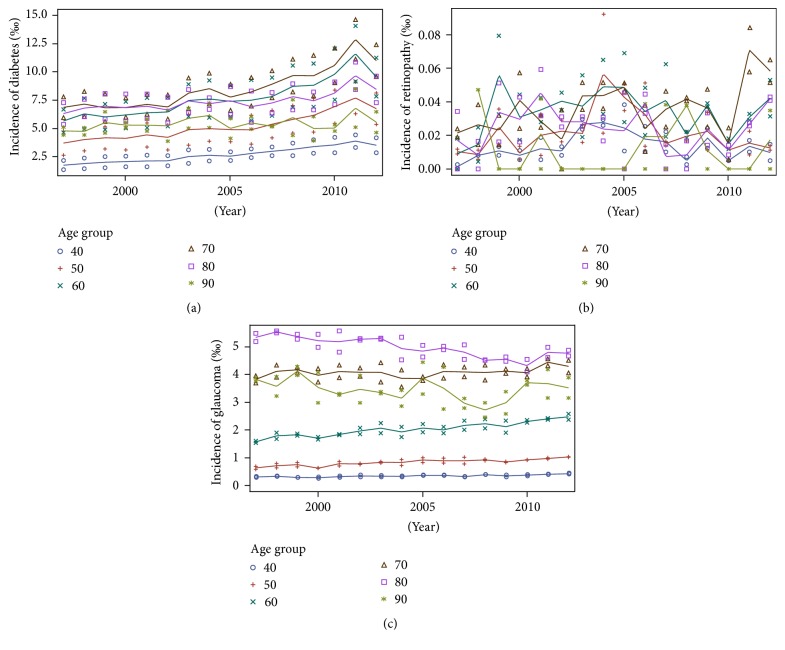
Incidence of diabetes mellitus, diabetic retinopathy, and glaucoma in the Danish population, in the period from 1996 to 2012, per 1000 individuals (‰). (a) Diabetes mellitus incidence. (b) Diabetic retinopathy incidence. (c) Glaucoma incidence.

The incidence of DM, DR, and glaucoma in the Danish population over the period from 1996 to 2012 is depicted in Figure 2. A constant number of new glaucoma cases per year were identified in the total period, whereas the amount of new DM cases per year appeared to increase in the same period.

### 3.3. Incidence Rates for DM and Glaucoma

**Figure 3:**
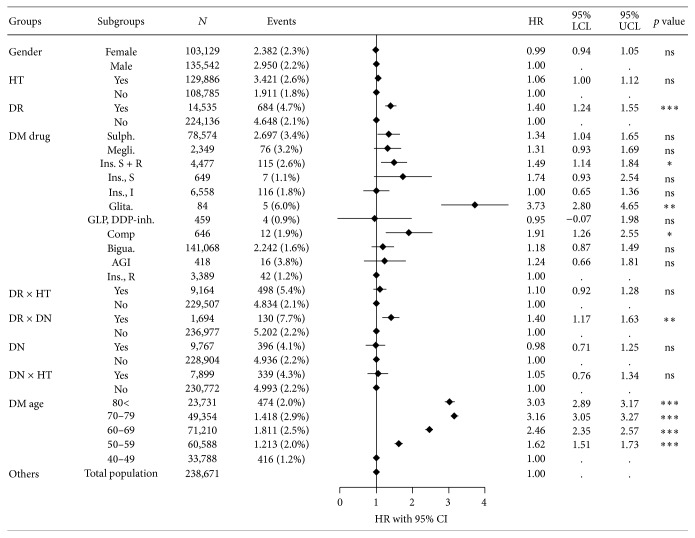
Hazard ratios for glaucoma development in patients treated with antidiabetic drugs. A range of confounding factors, comorbidity, concomitant medications factors, age, and gender are being adjusted for. The underlying data represents patients ≥ 40 years of age. For data on the total diabetic population, see Table 2. HR: hazard ratio; *N*: number of individuals; events: number of patients with glaucoma; CL: confidence limit. ^*∗*^Significant on the 5% level. ^*∗∗*^Significant on the 1% level. ^*∗∗∗*^Significant at the 0.1% level.

The results showed an association between DM and the increased risk of new-onset glaucoma (Table 2). The overall incidence rates per 100 person-years were 0.070 (95% CI 0.069–0.071) and 0.36 (95% CI 0.35–0.37) for the reference population and patients with DM, respectively. However, a common association with age or other confounding factors may be the cause of such an association. In particular, the risks of developing either condition increase with age (Figure 3), which can potentially explain this correlation. Therefore, we account for potentially confounding factors in a duration model, presented in the next subsection.

### 3.4. Duration Analysis

To exclude that increased incidence of glaucoma among patients treated with antidiabetic medication is simply caused by a common association with age or other potentially confounding factors, a duration model was implemented.

Table 1 shows a series of duration models accounting for various sets of potential covariates, namely, sex, age, and calendar year fixed effects. The duration models estimate the RR for developing glaucoma in patients treated with antidiabetic drugs in the Danish population in the period from 1996 to 2012. Column 1 presents the unconditional association between DM and glaucoma. It establishes that patients treated with antidiabetic drugs have a significantly higher risk of glaucoma compared to people who never redeemed prescriptions of antidiabetic drugs (RR = 5.13, *p* < 0.0001). Column 2 establishes that patients having DR have a significantly higher risk of glaucoma compared to people who do not have DR (RR = 4.69, *p* < 0.0001). Column 3 indeed shows that use of antidiabetic drugs (RR = 5.11, *p* < 0.0001) and DR (RR = 1.93, *p* < 0.0001) have an increased risk for glaucoma. The model further accounts in column 4 for the gender of the individuals and establishes that the RR of glaucoma in the DM and DR patients is still above unity (RR = 5.16, *p* < 0.0001 and RR = 1.54, *p* < 0.0001, resp.), with a significantly higher risk of glaucoma in women (RR = 1.35, *p* < 0.0001). Column 5 establishes that treatment with antidiabetic drugs is still associated with an increased risk of glaucoma while accounting for age (as five-year age group fixed effects) in addition to sex (RR = 1.81, *p* < 0.0001) and diagnosed DR (RR = 1.86, *p* < 0.0001). Finally, column 6 establishes that antidiabetic drugs are still significantly associated with glaucoma while accounting for calendar year fixed effects in addition to the other control variables (RR = 2.05, *p* < 0.0001).

### 3.5. Hazard Ratios of New-Onset Glaucoma in Patients with DM

Using multivariate Cox regression model analyses, all individuals treated with antidiabetic drugs were investigated for the HR and adjusted for age, sex, comorbidity, concomitant medications, all being factors which can potentially affect the risk of glaucoma.

In Table 2, the HR for a range of factors is shown for all individuals, as well as for patients ≥ 40 years of age. The latter regression results are illustrated in Figure 3. Overall, an increased HR for glaucoma is found in patients having DR, concomitant DR, and diabetic nephropathy and hypertension. Furthermore, we confirm the above reported substantial age-dependence, whereas the observed gender difference is not statistically significant in this specification. The results also show that patients treated with sulfonylureas, glitazone, and slow-acting insulin in combination with rapid-acting insulin have a significantly higher HR for developing glaucoma compared to patients treated with rapid-acting insulin analogues (Table 2). Following up on the reported increased HR in patients having concomitant antihypertensive medication, the differences in the type of antihypertensive medication in patients with DM were investigated (Table 2, columns 2 and 4). Indeed, the type of antihypertensive medication used has a significant effect, while a combination of BB with RAS analogues seems to have a proactive effect. Furthermore, RAS and other antihypertensive drugs have a significant increased effect, while BBs in combination with one other antihypertensive medicament have a tendency to lower the risk of glaucoma, although not significantly from that of having no hypertension at all.

## 4. Discussion

To the best of our knowledge, the present study is one of the largest studies investigating the association between glaucoma and DM, using data for an entire population over a sixteen-year period. We find an overall increased risk of glaucoma among patients with DM. This association remains evident when controlling for age, gender, retinopathy, and year-specific fixed effects.

Furthermore, we find that, in patients with DM, comorbidity with hypertension as well as presence of DR and/or joint complications with DR and nephropathy increases the risk of glaucoma. However, treatment with the antihypertensive combination of *β*-blockers and renin-angiotensin system inhibitors appears to be associated with a significantly lower hazard ratio for glaucoma onset in DM patients.

Other large population-based studies have also demonstrated an association between glaucoma and DM [10–12, 20, 21], whereas the largest case-control study and cohort study failed to find any association, explaining this lack of association by a possible referral bias [9, 22].

Among studies which supported the association, the Wisconsin study [12] found an odds ratio (OR) of 1.84 (95% CI, 1.09–3.11), while the study from Australia [10] found an OR of 2.12 (95% CI, 1.18–3.79), and the study from Rotterdam [11] found an OR of 3.11 (95% CI, 1.12–8.66). An independent study from Baltimore found no significant correlation with an OR of 1.03 (95% CI, 0.85–1.25) [9]. A recent cohort study from Scotland further was lacking to find an association between DM and glaucoma, when adjusting for age [16].

A number of studies have pointed to the possibility that DM may affect the vascular autoregulation of retina and the optic nerve and thereby promote the risk of DR and glaucoma [20, 23, 24]. The present study reports a significant correlation between DM and glaucoma, showing a higher risk of glaucoma in patients treated with antidiabetic medication. Adjusting for age and gender, the correlation is still significant. In addition, adjusting for all year-specific fixed factors, such as changes in public health policies or medical innovations that may affect the treatment of both diseases, does not change much the correlation between the two conditions.

Although our results indicate a strong association between use of DM drugs and the use of antiglaucomatous drugs, we cannot rule out the possibility that the observed association is affected by the fact that patients with DM may get eye diseases detected more often by routine clinical care compared to healthy individuals.

As an additional finding, the present study finds an increased risk of glaucoma among DM patients with DR and/or joint complications with DR and nephropathy. The mechanisms behind this association could simply be that these patients suffer from a more severe DM disease. Both DR and nephropathy are serious conditions that need intensive treatment. Furthermore, diabetic nephropathy is often treated with ACE inhibitor as well as lipid lowering treatment and aspirin.

A possible increased risk of glaucoma in patients with concomitant hypertension and DM was further investigated. Overall, we show that antihypertensive medication is associated with an increased risk of glaucoma in patients with DM. However, the combination of *β*-blocker and renin-angiotensin system inhibitors appears to lower the hazard ratio for glaucoma onset in DM patients. In general, inhibitors of the renin-angiotensin system (RAS) are a commonly used medicament in treatment of hypertension. In addition to regulate blood pressure, RAS is an active local system in the eye [25–28] (1–7) and ACE is found to be significantly higher in glaucomatous eyes [25]. The literature highlights the importance of particularly angiotensin II in the etiology of eye diseases. Some studies have shown that ACE inhibitor reduces the IOP and have a protective effect against glaucoma [29–31], but it has also been shown that the peptide angiotensin II is a modulator or transmitter in retinal neurophysiology. Thereby, an inhibition of ACE results in a decrease in angiotensin II that might cause disturbance of retinal neuronal function [28, 32]. Furthermore, a counterbalancing interaction between ACE II products and ACE-I has been suggested to be important [27, 33]. Our study indicates that inhibition of the RAS either increases the risk of glaucoma or reflects a more severe form of DM. However, we find that patients receiving a combination of RAS and BB have a significantly lower risk of glaucoma compared to DM patients without comorbidity with hypertension. One explanation for the decreased risk in patients treated with BB could be the intraocular pressure-lowering effect of this drug [34]. In our study, we reveal that RAS is generally positively associated with glaucoma, except in the combination with BB. We believe that this difference in associations can be due to either a preventive effect of BB or a synergistic effect of RAS and BB. Further studies are needed to disentangle these possible mechanisms. If this observed association is proven, it may suggest that RAS treated patients with DM would need further attention for diagnosis or treatment of glaucoma.

The main strength of our study is the use of comprehensive data resources covering a large population base, namely, the entire Danish population followed over 16 years. In particular, the National Danish Registry records 100% of all dispensed prescriptions in all pharmacies in Denmark, and, furthermore, all births, deaths, emigrations, and immigrations in Denmark. However, a limitation of the study is that we use prescriptions as the indicator of glaucoma and DM. In this matter, we are not able to conclude anything concerning the etiology or severity of the conditions.

In conclusion, this study reports an increased risk of glaucoma among patients treated with antidiabetic drugs. Furthermore, comorbidity with DR and the joint comorbidity with DR and/or diabetic nephropathy increase the hazard of getting glaucoma. Concomitant medications such as antihypertensive drugs can also increase the hazard for developing glaucoma. However, particular treatment with BB decreases the risk of glaucoma, while combination of BB and RAS conversely increases the risk.

## Figures and Tables

**Table 1:** Duration analysis. The table shows the relative risk for developing glaucoma in patients treated with antidiabetic drugs in the Danish population (1996–2012). The model controls for complication with DR and age as well as calendar year fixed effects (omitted from the table). RR: relative risk; CI: confidence interval; anti-DM drug: antidiabetic drug.

Model	1 RR (95% CI)	2 RR (95% CI)	3 RR (95% CI)	4 RR (95% CI)	5 RR (95% CI)	6 RR (95% CI)
Anti-DM drug *(Reference: no anti-DM drugs)*	5.13^*∗*^ (4.99–5.28)		5.11^*∗*^ (4.97–5.25)	5.16^*∗*^ (5.02–5.31)	1.81^*∗*^ (1.76–1.86)	2.05^*∗*^ (1.57–2.68)
DR *(Reference: no DR)*		4.69^*∗*^ (3.67–6.00)	1.93^*∗*^ (1.51–2.46)	1.97^*∗*^ (1.54–2.52)	1.86^*∗*^ (1.46–2.38)	1.82^*∗*^ (1.42–2.33)
Gender *(Reference: males)*				1.35^*∗*^ (1.33–1.37)	1.17^*∗*^ (1.15–1.18)	1.16^*∗*^ (1.15–1.18)

Age	No	No	No	No	Yes	Yes
Calendar year	No	No	No	No	No	Yes

^*∗*^ *p* < 0.001.

**Table 2:** Multivariable Cox regression model analyses showing the hazard ratios for glaucoma by antidiabetic drugs used in patients with DM. The effect of complications such as diabetic retinopathy and nephropathy was investigated, as well as the concomitant medications used, such as antihypertensive drugs. RAS: renin-angiotensin system; PE: parameter estimate; SE: standard error; HR: hazard ratio.

Model	Age ≥ 40 years				All ages			
	1		2		3		4	
	PE (SE)	HR	PE (SE)	HR	PE (SE)	HR	PE (SE)	HR
DR *(Reference: no DR)*	0.33^*∗∗∗*^ (0.08)	**1.4**	0.45^*∗∗∗*^ (0.04)	**1.57**	0.34^*∗∗∗*^ (0.07)	**1.4**	0.44^*∗∗∗*^ (0.04)	**1.55**
Diabetic nephropathy	−0.02 (0.14)	0.98	0.11^*∗*^ (0.05)	**1.12**	0.03 (0.13)	1.03	0.13^*∗∗*^ (0.05)	**1.14**
Hypertension *(Reference: no antihypertensives)*	0.06 (0.03)	1.06			0.11^*∗∗*^ (0.03)	**1.12**		
Gender *(Reference: males)*	−0.01 (0.03)	0.99	−0.01 (0.03)	0.99	0 (0.03)	1	0 (0.03)	1
Nephropathy × DR	0.34^*∗∗*^ (0.12)	**1.4**			0.34^*∗∗*^ (0.11)	**1.41**		
Nephropathy × hypertension	0.05 (0.15)	1.05			0.01 (0.14)	1.01		
DR × hypertension	0.1 (0.09)	1.1			0.08 (0.09)	1.08		

Biguanides	0.17 (0.16)	1.18	0.17 (0.16)	1.19	0.24 (0.14)	1.27	0.24 (0.14)	1.28
Combination	0.64^*∗*^ (0.33)	**1.91**	0.66^*∗*^ (0.33)	**1.93**	0.71^*∗*^ (0.32)	**2.04**	0.73^*∗*^ (0.32)	**2.07**
GLP-1-analogues and DDP-IV-inhibitors	−0.05 (0.52)	0.95	−0.05 (0.52)	0.96	0.26 (0.47)	1.3	0.27 (0.47)	1.31
Glitazones	1.32^*∗∗*^ (0.47)	**3.73**	1.31^*∗∗*^ (0.47)	**3.72**	1.36^*∗∗*^ (0.47)	**3.88**	1.36^*∗∗*^ (0.47)	**3.89**
Insulin and insulin analogues (slow)	0.55 (0.41)	1.74	0.57 (0.41)	1.77	0.47 (0.4)	1.59	0.48 (0.4)	1.62
Insulin and insulin analogues (slow combined with rapid)	0.4^*∗*^ (0.18)	**1.49**	0.4^*∗*^ (0.18)	**1.49**	0.47^*∗∗*^ (0.16)	**1.59**	0.47^*∗∗*^ (0.16)	**1.6**
Insulin and insulin analogues (intermediate)	0 (0.18)	1	0.01 (0.18)	1.01	0.12 (0.16)	1.13	0.13 (0.16)	1.14
Meglitinides	0.27 (0.19)	1.31	0.27 (0.19)	1.31	0.35^*∗*^ (0.18)	**1.43**	0.36^*∗*^ (0.18)	**1.43**
Sulphonylurea	0.29 (0.16)	1.34	0.3 (0.16)	1.35	0.4^*∗∗*^ (0.14)	**1.49**	0.4^*∗∗*^ (0.14)	**1.5**
*α*-Glucosidase inhibitors	0.21 (0.29)	1.24	0.21 (0.29)	1.24	0.33 (0.28)	1.4	0.34 (0.28)	1.4

RAS + *β*-blocker			−0.14^*∗∗*^ (0.06)	**0.87**			−0.09 (0.06)	0.92
RAS + antiadrenergic			0.52^*∗∗*^ (0.18)	**1.68**			0.56^*∗∗*^ (0.18)	**1.75**
RAS + Ccb			0.19^*∗∗∗*^ (0.05)	**1.21**			0.23^*∗∗∗*^ (0.05)	**1.25**
RAS + Diu			0.16^*∗∗∗*^ (0.04)	**1.18**			0.21^*∗∗∗*^ (0.04)	**1.24**
RAS + Vas			0.23 (0.13)	1.26			0.24^*∗*^ (0.13)	**1.28**
RAS *β*-blocker + 1 other			−0.03 (0.07)	0.97			0.01 (0.07)	1.01
RAS + (other ≥ 2)			0.11 (0.09)	1.12			0.17^*∗*^ (0.08)	**1.18**
*β*-Blocker + other antihyp. drugs ≥ 1			−0.03 (0.05)	0.97			0.03 (0.05)	1.03
Other antihyp. drugs ≥ 2			0.09 (0.05)	1.09			0.15^*∗∗*^ (0.05)	1.16

Age 50–59 years *(ref 40*–*49)*	0.48^*∗∗∗*^ (0.06)	**1.62**	0.49^*∗∗∗*^ (0.06)	**1.63**				
Age 60–69 years *(ref 40*–*49)*	0.9^*∗∗∗*^ (0.06)	**2.46**	0.91^*∗∗∗*^ (0.06)	**2.48**				
Age 70–79 years *(ref 40*–*49)*	1.15^*∗∗∗*^ (0.06)	**3.16**	1.16^*∗∗∗*^ (0.06)	**3.19**				
Age > 80 years *(ref 40*–*49)*	1.11^*∗∗∗*^ (0.07)	**3.03**	1.12^*∗∗∗*^ (0.07)	**3.06**				

Age 21–40 years *(ref < 21)*					1.07^*∗∗*^ (0.3)	**2.91**	1.08^*∗∗*^ (0.3)	**2.95**
Age 41–60 years *(ref < 21)*					2.33^*∗∗∗*^ (0.3)	**10.25**	2.34^*∗∗∗*^ (0.3)	**10.39**
Age 61–80 years *(ref < 21)*					2.96^*∗∗∗*^ (0.3)	**19.25**	2.98^*∗∗∗*^ (0.3)	**19.6**
Age > 80 years *(ref < 21)*					3.04^*∗∗∗*^ (0.3)	**20.8**	3.06^*∗∗∗*^ (0.3)	**21.23**

Number of individuals		238,671	238,671		277,266		277,266	

^*∗*^ *p* < 0.05,  ^*∗∗*^ *p* < 0.01, and  ^*∗∗∗*^ *p* < 0.001.
